# Manuscript publication of abstracts presented at gynecologic surgery societies’ annual meetings

**DOI:** 10.1007/s00404-024-07865-9

**Published:** 2025-01-17

**Authors:** Kasey C. Fitzsimmons, Kacey M. Hamilton, Rebecca J. Schneyer, Shlomi Toussia-Cohen, Shannon Fan, Nikki R. Farsa, Gabriel Levin, Kelly N. Wright, Raanan Meyer

**Affiliations:** 1 David Geffen School of Medicine, University of California Los Angeles (UCLA), Los Angeles, CA USA; 2 Division of Minimally Invasive Gynecologic Surgery, Department of Obstetrics and Gynecology, Cedars Sinai Medical Center, Los Angeles, CA USA; 3 Department of Obstetrics and Gynecology, Sheba Medical Center, Ramat-Gan, Israel; 4 Faculty of Medicine, Tel-Aviv University, Tel-Aviv, Israel; 5 Department of Obstetrics and Gynecology, Cedars Sinai Medical Center, Los Angeles, CA USA; 6 University of California, San Diego, CA USA; 7 Department of Gynecologic Oncology, Jewish General Hospital, McGill University, Quebec, Canada; 8 The Department of Gynecologic Oncology, Hadassah Medical Center, Faculty of Medicine, Hebrew University of Jerusalem, Jerusalem, Israel

**Keywords:** Abstract to full-text publication, Journal society, Gynecologic surgery, H-index, Obstetrics and gynecology

## Abstract

**Purpose:**

To study characteristics and identify factors associated with full manuscript publication of oral abstracts presented at gynecologic surgery societies’ annual meetings.

**Study design:**

We reviewed all oral abstracts presented at four major gynecologic surgery meetings in 2018. Oral abstracts subsequently published as peer-reviewed manuscripts were compared to those that were not published. Descriptive statistical analysis and multivariable regression analyses were conducted to identify factors associated with peer-reviewed manuscript publication.

**Results:**

A total of 396 oral presentation abstracts from the four nationally recognized gynecologic societies were identified. The overall journal publication rate was 47.4% (188/396). The rate of publication of oral abstracts was 35.1% (72/205) for those presented at AAGL, 73.8% (62/84) for AUGS, 53.2% (42/79) for SGO and 42.9% (12/28) for SGS. In multivariable regression analysis, last author’s H-index [aOR 95% CI 1.02 (1.00–1.03)], academic center affiliation [aOR 95% CI 2.29 (1.20–4.37)], and randomized controlled trials [aOR 95% CI 2.47 (1.12–5.47)] were associated with journal publication. Of the published articles, the median time to publication was 3.0 years [1.0–5.0], the median journal impact factor was 3.9 [1.8–4.8], the median relative citation ratio was 1.0 [0.4–1.9], and the median number of citations per year was 2.0 [1.0–4.1].

**Conclusions:**

In the field of gynecologic surgery, several factors, including the last researcher’s H-index, academic affiliation, randomized controlled trial design and type of societal meeting are associated with increased odds of an oral abstract ultimately reaching full manuscript peer-reviewed publication. These findings can serve researchers in the fields of gynecologic surgical subspecialties.

## What does this study add to the clinical work

**Table:** 

This is the first study that identifies factors associated with manuscript publication of abstracts presented at gynecologic surgery societies’ annual meetings.	

## Introduction

Scientific meetings, many established by national medical societies, are key forums where leaders, industries, educators and trainees in their chosen field gather together to share knowledge. Abstracts presented in annual meetings are often viewed as a prelude to a subsequent full- manuscript and peer-reviewed publication, the cornerstone of scientific publishing integrity [1]. Many abstracts presented at annual meetings remain unpublished despite the importance of peer-reviewed publication. This lack of peer-reviewed medical knowledge could have long-term consequences, such as influencing research quality, monetary loss, and quality of health care delivery [2]. Furthermore, peer-reviewed publications are a critical metric for academic promotion, hiring practices, funding, and grant decisions [3]. In the field surgical gynecology, there remains limited knowledge about the factors that may influence abstract to full-text publication of research. Prior studies, specific to urogynecology and gynecologic oncology, have looked at society full-text conversion rates, but there has yet to be a more comprehensive analysis of multiple gyn-related societies and the larger impact they have on publication rates of surgical gynecology broadly [4–10].

Therefore, we aimed to study characteristics and identify factors associated with full manuscript publication of oral abstracts presented at the four largest gynecologic surgery societies in the United States: American Association of Gynecologic Laparoscopists (AAGL), American Urogynecologic Society (AUGS), Society of Gynecologic Oncology (SGO), and Society of Gynecologic Surgeons (SGS).

## Materials and methods

This was a cross-sectional study. We reviewed all oral abstracts presented at four national gynecologic surgical societies at their 2018 annual meetings: AAGL, AUGS, SGO, and SGS [11–14]. The year 2018 was chosen to limit any delay or impact the COVID-19 pandemic may have had on abstract submissions and journal acceptance.

Abstracts presented at these annual meetings were identified via their society. The following variables were collected from the identified abstracts as they represent factors that may influence researcher power to publish: society name, study’s state of origin, first and last author gender, first and last author h-index, author affiliation [university hospital, National Institute of Health (NIH), community], study design [observational, randomized controlled trial (RCT), meta-analysis/systematic review], number of study participants, and single versus multicenter study. Any ambiguity to the above variables was explored using LinkedIn and healthcare systems websites to confirm variables such as researcher gender, affiliation, or name discrepancies. The h-index of the abstracts’ first and last authors were retrieved from the Web of Science database.

To identify abstracts accepted for full manuscript journal publication, we searched the following databases: PubMed, EMBASE, and Google Scholar, from January 2018 to November 2023. The list of abstracts was split into four groups, and four reviewers performed a search independently (KF, STC, NF, SF). A second review was conducted by the same four reviewers but on a different split of the list of abstracts. Inconsistencies between presented and published abstracts were assessed independently by the four evaluators, then reached a consensus with the following guidelines.

To identify published abstracts, we used combinations of author names, titles, and keywords in the databases listed above. Published titles with altered titles from the abstract were accepted if the published author list matched the abstract’s author list, the study setting, design and results were similar to those reported in the abstract. Published work with duplicate titles as the abstract but with differing author lists was accepted if the first and last author matched the abstract, as well as the study setting, design, and results. Only manuscripts accepted to peer-reviewed journals with impact factors were included.

Once publications were identified, we collected the following variables: time (in years) to publication, journal impact factor (from Web of Science), relative citation ratio (RCR), and citations per year (from the Journal Citation Reports database). RCR is an article-level metric that provides a field-normalized ratio and is not dependent on time [15].

We compared published to unpublished oral abstracts to identify factors associated with full-manuscript publication. We further analyzed the characteristics of the published abstracts.

### Statistical analysis

We performed a descriptive analysis using a Chi-square test and Fisher’s exact test as appropriate. Mann–Whitney *U* test was used to analyze continuous variables. Categorical variables were reported as median with interquartile range and continuous variables as proportions. Multivariable regression analysis was conducted to identify independent parameters associated with journal publication. The regression analysis model included factors found to be statistically significant in the univariate analysis. We performed two multivariable regression analyses, one of which included the four surgical societies, to account for the different characteristics of the annual meetings. A two-sided *p* value < 0.05 was considered statistically significant. The results are reported as adjusted odds ratio (aOR) and 95% confidence interval (CI). Statistical analyses were performed using Software Package for Statistics and Simulation (IBM SPSS version 27, IBM Corp, Armonk, NY).

### Ethical approval

As the study used deidentified, publicly available data, it was deemed exempt by the Institutional Review Board of the Cedars Sinai Medical Center (#00003363).

## Results

A total of 396 oral presentation abstracts from the four nationally recognized gynecologic societies were identified: 205 from AAGL, 84 from AUGS, 79 from SGO, and 28 from SGS. The characteristics of published and unpublished abstracts are outlined in Table 1. The overall journal publication rate was 47.4% (188/396). The rate of publication of oral abstracts was 35.1% (72/205) for those presented at AAGL, 73.8% (62/84) for AUGS, 53.2% (42/79) for SGO and 42.9% (12/28) for SGS. A higher proportion of abstracts from AUGS and SGO were published rather than unpublished, whereas AAGL and SGS had more unpublished abstracts compared to published abstracts. The number of authors assigned to an individual abstract was higher in the published group compared to the unpublished group (median authors 5.5 vs. 4.0, *p* < 0.001). The percentage of female authorship, both first author and last author, was higher in the published group compared to the unpublished group (66.5% vs. 58.2%, *p* = 0.048 and 52.7% vs. 38.9%, *p* = 0.005, respectively). The author’s h-index, both first and last author, was higher in the published group compared to the unpublished group (median 7.0 vs. 4.0 and 17.0 vs. 9.5, respectively, *p* < 0.001). Abstracts written by authors with academic affiliations were associated with higher proportion of publication (87.8% vs. 68.3%, *p* < 0.001). The median number of study participants was higher in the published group compared to the unpublished group (170.5 vs 111.0, *p* = 0.010). Most of the published abstracts were observational studies (79.3%), followed by RCTs (17.0%) and systematic reviews, meta-analyses (3.7%). When comparing submitted abstracts for observational studies to those of RCTs, more RCTs were accepted for publication versus not accepted (17.0% vs. 7.7%, *p* = 0.002) whereas the oppositive occurred with observational studies (79.3% vs. 91.3%), likely representing the strength of controlled trials compared to observational studies and the higher number of observational studies submitted overall [15]. A majority of the multicenter studies were published (41.5% vs. 26.9%), while a higher percentage of single-center studies remained unpublished (58.5% vs. 73.1%, *p* = 0.003).

**Table 1:** Comparison of published and unpublished characteristics of society meetings oral presentations

	Published *n* = 188	Unpublished *n* = 208	*p* value
Society meeting name			< 0.001
American association of gynecologic laparoscopists	72 (38.3)	133 (63.9)	
American urogynecologic society	62 (33.0)	22 (10.6)	
Society of gynecologic oncology	42 (22.3)	37 (17.8)	
Society of gynecologic surgeons	12 (6.4)	16 (7.7)	
Studies originating in the United States	139 (73.9)	146 (70.2)	0.407
Number of authors	5.5 [4.0, 7.0]	4.0 [3.0, 6.0]	< 0.001
Female first author	125 (66.5)	121 (58.2)	0.048
Female last author	99 (52.7)	81 (38.9)	0.005
First author’s H-index	7.0 [3.0–13.0]	4.0 [1.0–10.0]	< 0.001
Last author’s H-index	17.0 [9.0–28.0]	9.5 [3.0–21.0]	< 0.001
First author’s affiliation			< 0.001
Non-academic center	23 (12.2)	66 (31.7)	
Academic center	165 (87.8)	142 (68.3)	0.010
Number of participants in the study	170.5 [65.0–587.3]	111.0 [26.0–408.5]	
Study design			0.002
Observational	149 (79.3)	190 (91.3)	
Randomized controlled trial	32 (17.0)	16 (7.7)	
Meta-analysis or systematic review	7 (3.7)	2 (1.0)	
Number of centers			0.003
Single center	110 (58.5)	152 (73.1)	
Multicenter study	78 (41.5)	56 (26.9)	

Data are *n* (%) or median [Interquartile range]

Table 2 displays the results of the multivariable logistic regression analysis. The following variables were independently associated with journal publication: last author’s H-index [aOR 95% CI 1.02 (1.00–1.03)], academic hospital affiliation [aOR 95% CI 2.29 (1.20–4.37)], and RCTs compared to other study designs [aOR 95% CI 2.47 (1.12–5.47)].

**Table 2:** Multivariable regression analysis of factors associated with publication of oral presentations excluding society

	Adjusted odds ratio	95% confidence interval	*p* value
Number of authors	1.03	0.94–1.13	0.518
Female first author	1.06	0.61–1.83	0.846
Female last author	1.48	0.89–2.45	0.133
First author’s H-index	1.00	0.98–1.03	0.770
Last author’s H-index	1.02	1.00–1.03	0.039
First author’s affiliation			
Non-academic center or other	Ref	–	–
Academic center	2.29	1.20–4.37	0.012
Number of participants in the study	1.00	1.00–1.00	0.328
Study design			
Observational or other	Ref	–	
Randomized controlled trial	2.47	1.12–5.47	0.026
Number of centers			
Single center	Ref	–	
Multicenter study	1.30	0.76–2.20	0.336

Table 3 displays the results of the multivariable logistic regression analysis including type of society meeting. Compared to AAGL presentation, AUGS presentation was associated with full-manuscript publication [aOR 95% CI 2.84 (1.32–6.10)]. Last author’s H-Index and academic center affiliation remained independently associated with publication, while RCT design was no longer independently associated with publication.

**Table 3:** Multivariable regression analysis of factors associated with publication of oral presentations including society

	Adjusted odds ratio	95% confidence interval	*p* value
Society meeting name			
American association of gynecologic laparoscopists	Ref	–	–
American urogynecologic society	2.84	1.32–6.10	0.008
Society of gynecologic oncology	0.51	0.24–1.10	0.087
Society of gynecologic surgeons	2.45	0.70–8.60	0.163
Number of authors	1.06	0.96–1.17	0.242
Female first author	0.94	0.53–1.66	0.832
Female last author	1.26	0.74–2.15	0.390
First author’s H-index	1.01	0.99–1.04	0.431
Last author’s H-index	1.02	1.00–1.04	0.024
First author’s affiliation			
Non-academic center or other	Ref	–	–
Academic center	2.12	1.08–4.19	0.048
Number of participants in the study	1.00	1.00–1.00	0.306
Study design			
Observational or other	Ref	–	
Randomized controlled trial	1.39	0.57–3.37	0.473
Number of centers			
Single center	Ref	–	
Multicenter study	1.51	0.87–2.61	0.142

Table 4 displays the characteristics of the 188 published abstracts. The median time to publication was 3.0 years [1.0–5.0]. The median journals’ impact factor was 3.9 [1.8–4.8]. The median RCR was 1.0 [0.4–1.9]. The median number of citations per year was 2.0 [1.0–4.1].

**Table 4:** Characteristics of published manuscript

	Published *n* = 188
Years to publication	3.0 [1.0–5.0]
Journal impact factor	3.9 [1.8–4.8]
Relative citation ratio	1.0 [0.4–1.9]
Citations per year	2.0 [1.0–4.1]

Data are median [Interquartile range]

## Discussion

When studying oral abstracts presented at four surgical gynecologic annual meetings, the overall proportion of journal publications was 47.4%. Last author’s higher H-index, academic hospital affiliation and RCTs were independently associated with increased adjusted odds of journal publications. When including type of society meeting, AUGS as compared to AAGL presentation was associated with increased adjusted odds of publication.

Almost half of the presented oral abstracts reached a full-length publication. In line with our results, the conversion rate of major generalist- and fellowship-associated OBGYN society meeting abstracts in 2013 was 54% [5]. In comparison with other surgical subspecialty journals, gynecologic surgery journals’ conversion proportion was lower than plastic surgery (65%) and orthopedic surgery (60.2%) [16–18]. A large Cochrane review of biomedical journal publications which examined more than 300 K abstracts, found a 31.9% publication rate [19]. It would appear that while gynecologic surgery has not shown the publishing prowess that other surgical subspecialties tend to display, the field does tend to publish more than the average publication rates of biomedical journals, and is in line with other generalist OBGYN journals. One possible explanation for this could be that our study specifically incorporated oral abstracts, and did not include other types of abstracts. Assuming that oral presentations are associated with higher odds of publication, it is possible that the surgical gynecology publication conversion rate may actually be lower than 47.4% if all abstract types were to be examined [16–19]. Another possible external variable could be that the COVID-19 pandemic has been shown to affect the publication rate of non-COVID-19 abstracts [20]. However, it is not likely we have underestimated the publication rate, as the journal abstracts were given close to six years to publish at the time this study was conducted, and as other subspecialty surgical abstracts have shown a publication rate as high as 90% within four years of the meeting [2, 18]. We can, therefore, deduce that the conversion rate for gynecologic surgery journal abstracts into full-text publications likely remains around 50%.

The last author’s H-index was identified as a predictor of journal publication, with authors who published their abstracts having an H-index almost double those who did not publish. The H-index is the most famous researcher quality metric and represents an essential reference for nearly all physicians [21]. It is defined by a researcher’s number of published articles and the number of citations each publication received. Currently, there remains a lack of insight into an individual researcher’s H-index on their future publication likelihood. Furthermore, we did not identify prior publication on the association of the H-index and publication of abstracts in OBGYN. Our results support the validity of the H-index for the last author but not for the first author. One explanation for this discrepancy is that the researcher’s scientific age strongly influences the H-index [22]. Consequently, younger researchers, those more likely to be first authors, are more likely to have lower H-index values because of the lower number of published papers and lower number of citations. A future study exploring the use of more inclusive alternatives to the H-index, including the G-index, AR-index, P-index, academic trace, and I3, and their impacts on gynecologic surgery publishing rates would be needed to explore the differences between the first and last authors [23]. Additionally, it would be interesting to further explore the relationship between the H-index and female authorship within the field of OBGYN specialty; especially given that our study found female authorship as associated with higher odds of publication in the univariate analysis.

We found that academic-affiliated authors had over double the odds of publishing their oral abstracts compared to their non-academic affiliated counterparts. This finding is in line with prior publications [2, 18]. Academic institutions have advantages, including larger and more diverse sample sizes, multi-site study subject recruitment, and potential for inter-disciplinary collaboration between researchers specifically recruited to academic centers [2, 18]. Considering the above, non-academic affiliated researchers interested in publishing their results may collaborate with academic institutions to increase the chance of full manuscript publication.

RCTs were associated with an almost 2.5-fold higher aOR of publication compared to other study design in the multivariable analysis excluding societies. This finding is not surprising given that RCTs are considered as the pinnacle of evidence as long as they are well designed and conducted [24]. Our results are in line with a prior Cochrane systematic review that examined factors associated with publication of biomedical meeting abstracts reported a 1.21 relative risk for publication of RCTs [19]. In contrast, a study of abstracts presented at representative OBGYN generalist and all subspecialty meetings with fellowship affiliation found a non-significant 1.7 aOR for publication of RCTs. Another study on publication of abstracts presented at SGO over 11 years has not identified an independent association between RCTs and publication, possibly due to limited sample size [5, 6]. Of note, the above-mentioned studies included both posters and oral abstracts, possibly accounting for the varying results.

Abstracts presented at the AUGS meeting were associated with higher odds of publication compared to the AAGL annual meeting. Of note, there were 60% fewer oral abstracts at AUGS. Possibly, the threshold for oral presentation acceptance was higher in the AUGS meeting, accounting for the observed differences [12]. We did not observe differences between AAGL and SGO or SGS odds of publications. The smaller sample size in the latter two may account for this lack of statistical difference. Of course, one must interpret comparisons between subspecialty journals with a degree of skepticism given that their subject matter are fundamentally different and separate journals may have different standards for publication.

The time to publication in our study is consistent with other publications within the general OBGYN specialty, with an average of 25.6 months to full-manuscript publication [19]. We found a median RCR of 1.0 amongst the full-text published surgical gynecology abstracts, similar to the median in the field. However, that number may be an underestimation; as of the 188 published manuscripts, 70 were published in 2023, limiting the time interval to assess the RCR. The median journals’ impact factor was 3.9, higher than 2.7, the median impact factor for journals in the field of OBGYN, according to Web of Science Journal Citation Report [25]. This finding suggests that manuscripts initially presented at annual meetings may be of higher quality compared to those that were not presented, and consequently accepted in higher impact factor journals.

Several limitations of this study should be acknowledged. First, we analyzed one year of oral abstracts and, therefore, cannot report on trends over consecutive years. Additionally, while every effort was made to identify published abstracts, it is possible that some abstracts were published in non-traditional venues not included in our search criteria. However, the number of omissions is likely small given our effort to search several databases, including PubMed, Google Scholar, and EMBASE. Moreover, an unknown number of manuscripts are submitted to journals directly, arrive at full publication without former presentation at meetings, and are not represented in this study. We could not account for the factors associated with these publications in the current study. Finally, although we took measures to mitigate its effect, the COVID-19 pandemic may have impacted the publication rate of the included abstracts.

A strength of our study is that its design sought to include factors to abstract publication not previously explored, such as H-index and its effects on publication likelihood. The evaluation of gynecologic surgery societies meetings is another strength of this study, allowing for an in-depth analysis of their publication characteristics.

In conclusion, in the field of gynecologic surgery, several factors, including the last researcher’s H-index, academic affiliation, RCT design and society surgical meeting type, are independently associated with publication of peer-reviewed manuscripts. From a practical standpoint, this study could be used by researchers to enhance the odds of full manuscript publication. Our results help expand upon and improve the understanding of how research abstracts presented at annual society meetings can be optimized for publication.

## Data Availability

The data that support the findings of this study are available from the corresponding author upon reasonable request.

## Abbreviations

AAGL: American association of gynecologic laparoscopists
AUGS: American urogynecologic society
RCR: Relative citation ratio
SGO: Society of gynecologic oncology
SGS: Society of gynecologic surgeons
